# Ayurvedic Management of Post-hysterectomy Osteoporosis: A Case Report

**DOI:** 10.7759/cureus.108840

**Published:** 2026-05-14

**Authors:** Yogita Pole, Amit Nakanekar

**Affiliations:** 1 Department of Kayachikitsa, Government Ayurved College and Hospital, Nagpur, Nagpur, IND

**Keywords:** ayurveda, basti, case report, osteoporosis, post-hysterectomy menopause

## Abstract

Osteoporosis is a systemic metabolic bone disorder characterized by decreased bone mass and microarchitectural deterioration. These changes increase fragility and fracture risk. The condition is seen particularly in postmenopausal and post-hysterectomy women. Hormone decline-related bone loss is often linked to chronic musculoskeletal pain, functional limitation, and reduced quality of life. Long-term use of modern antiresorptive therapies, though effective, may be limited by tolerability concerns and adherence challenges. This creates a rationale for safer, multimodal conservative approaches. A 55-year-old female with a history of hysterectomy in 2017 presented with a three-month history of generalized musculoskeletal pain predominantly affecting the knees and small joints of the hands, accompanied by thigh discomfort and functional limitation, and associated with generalized weakness, increased fatigue, and reduced mobility, prompting evaluation for underlying metabolic bone disease. She first reported to the OPD on February 4, 2025. Baseline evaluation showed low serum calcium (6.7 mg/dL), and bone density screening using quantitative ultrasound (QUS) of the right tibia showed a T-score of -2.6, which may suggest low bone density, including osteoporosis or possible osteomalacia. She received structured Ayurvedic integrative management over six months, with a short treatment gap due to self-discontinuation and later resumption. Clinically significant improvement was observed across validated outcome measures, including pain reduction, improvement in functional disability, and resolution of exertional limitation. Follow-up assessment demonstrated correction of hypocalcemia and improvement in QUS-based parameters, along with sustained symptomatic relief. This case describes symptomatic and biochemical improvement in a patient receiving an Ayurvedic intervention, with concurrent changes in QUS-based parameters. However, these findings are derived from a single case and a screening modality; hence, despite cautious interpretation, this observation generates a research question to evaluate the Ayurveda-based approach for improvement in bone mineral density or underlying mechanisms. The observations are hypothesis-generating and warrant further investigation using standardized interventions, dual-energy X-ray absorptiometry-based assessment, comprehensive metabolic evaluation, and controlled study designs to establish clinical efficacy, safety, and biological relevance.

## Introduction

Osteoporosis is a systemic skeletal disorder. It is characterized by reduced bone mass, microarchitectural deterioration, and increased fracture risk [1]. It affects about 200 million women worldwide. Prevalence rises from about 23.1% overall to more than 35.3% in postmenopausal groups [2]. The National Osteoporosis Risk Assessment study found osteopenia in 39.6% and osteoporosis in 7.2% of women aged ≥50 years without prior diagnosis, highlighting the clinical burden of this condition [3].

Hysterectomy has been associated with the risk of secondary osteoporosis, primarily due to impaired ovarian reserve and earlier estrogen deficiency. These changes lead to greater bone resorption and reduced bone formation. In cases where the ovaries are preserved, as in abdominal hysterectomy with ovarian conservation, the risk of accelerated bone loss is generally lower compared with bilateral oophorectomy, where abrupt estrogen deficiency occurs. However, some studies suggest that even with ovarian conservation, ovarian function may decline gradually over time, potentially contributing to an earlier onset of menopause and reduced bone mineral density [4]. Cohort data show a 1.3-1.7-fold increased risk of osteoporosis after hysterectomy. Substantial cases occur within seven years of surgery [5]. Current pharmacologic therapies are effective but are mainly antiresorptive. Their use is limited by long-term tolerability and adherence issues. This has led to interest in complementary and integrative approaches.

In Ayurveda, osteoporosis is conceptually correlated with Asthi-Kshaya (~loss of bone tissue), a condition associated with vitiation of Vata (one of the three doshas in Ayurveda, associated with movement and regulation). Progressive skeletal degeneration is attributed to tissue depletion and metabolic dysregulation. Some experimental studies have suggested that certain Ayurvedic interventions may have osteoprotective potential. However, clinical evidence remains limited, and proposed mechanisms such as gut-bone interactions or receptor-mediated pathways are largely theoretical and not yet established in clinical settings.

Therefore, we report a clinically documented case of post-hysterectomy osteoporosis that showed clinical improvement in symptoms and QUS-based bone density parameters following an integrative Ayurvedic therapeutic approach. This report aims to describe the clinical course and generate a hypothesis for future research, rather than to establish efficacy or definitive mechanisms.

## Case presentation

Patient information

A 55-year-old female presented to the OPD on February 4, 2025, with a three-month history of generalized musculoskeletal pain, predominantly involving the knees and small joints of the hands. The symptoms included thigh discomfort, generalized weakness, fatigue, and reduced mobility. The pain was severe at presentation (Visual Analogue Scale (VAS) 8/10) and resulted in significant functional limitations in daily activities.

She had undergone an abdominal hysterectomy with ovarian conservation in 2017 at Matru Sewa Sangh Hospital, Nagpur, for abnormal uterine bleeding. She attained menarche at 13 years of age and subsequently reached menopause at 43 years. There was no history of diabetes mellitus, hypertension, thyroid disorders, or other known endocrine conditions.

Dietary history revealed a mixed diet, including bakery products and milk, with frequent consumption of paryushita ahara (food prepared earlier and consumed after prolonged storage or loss of freshness), suggesting suboptimal dietary quality. The patient had taken calcium and vitamin D supplementation intermittently before presentation. The history of sunlight exposure was not systematically evaluated.

Physical activity was markedly reduced due to pain, and she experienced difficulty performing routine activities despite being a caregiver for physically challenged children. There was no history of prior fragility fractures, smoking, alcohol consumption, or long-term corticosteroid use. She did not report any prior diagnosis of rheumatoid arthritis or other systemic inflammatory disorders.

Clinical findings

**Figure 1:**
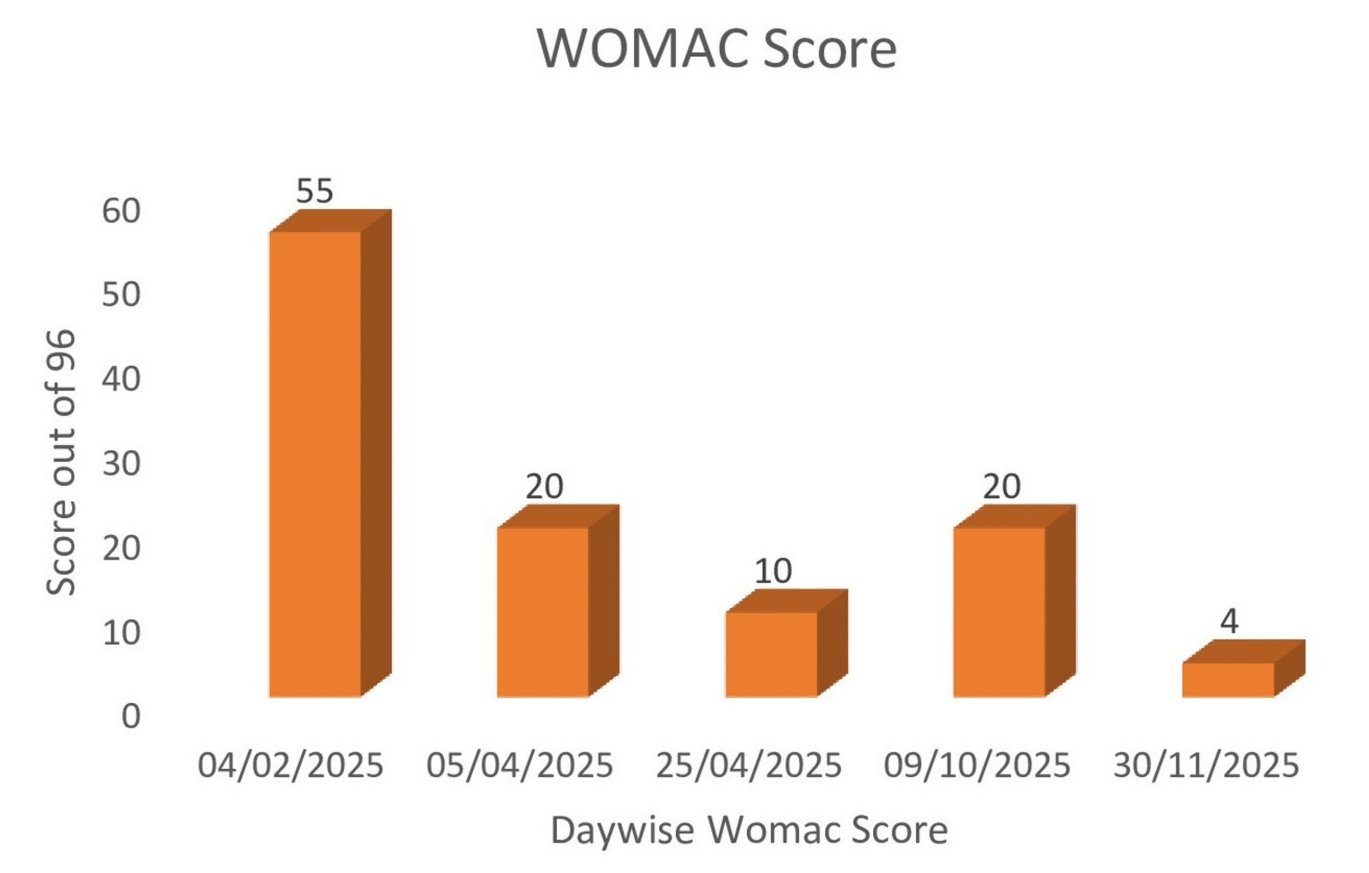
WOMAC score over time The figure demonstrates sequential changes in the WOMAC score during the treatment and follow-up period. A progressive reduction in the WOMAC score was observed from 55 at baseline (February 4, 2025) to 4 at final follow-up (November 30, 2025), indicating improvement in pain, stiffness, and functional disability during the course of integrative management. WOMAC, Western Ontario and McMaster Universities Osteoarthritis Index

**Figure 2:**
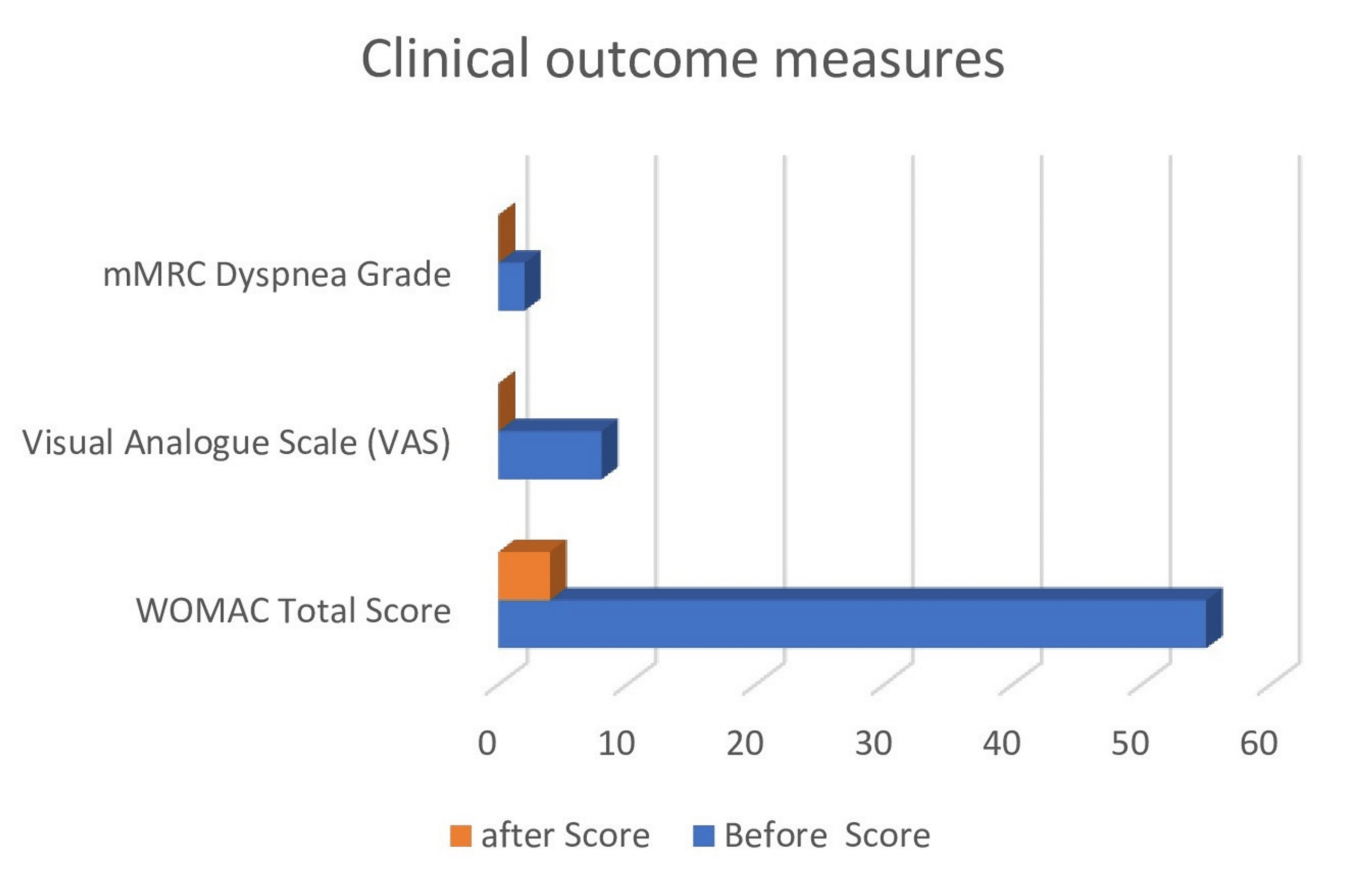
Clinical outcome measures The figure compares baseline and follow-up clinical outcome measures, including the WOMAC, VAS, and mMRC dyspnea grade. Reduction in all scores at follow-up indicates improvement in pain, functional disability, and associated symptoms during the course of integrative management. mMRC, modified Medical Research Council; VAS, Visual Analogue Scale; WOMAC, Western Ontario and McMaster Universities Osteoarthritis Index

On examination, her vital signs were normal: blood pressure 110/80 mmHg, pulse 78/min, respiratory rate 18/min, and peripheral oxygen saturation (SpO₂) 98% on room air. She was conscious, oriented, and hemodynamically stable. The musculoskeletal examination showed bilateral knee joint tenderness and pain with movement. There was also tenderness in the interphalangeal joints of both hands, without deformity or inflammation. She had difficulty with prolonged standing, climbing stairs, and gripping. This resulted in significant baseline disability (Western Ontario and McMaster Universities Osteoarthritis Index (WOMAC) 55 [6], VAS 8/10, and modified Medical Research Council (mMRC) dyspnea grade 2 [7]) (Figure 1, Figure 2).

Diagnostic assessment

The diagnosis was based on clinical presentation, biochemical findings, and bone density screening. The patient presented with severe symptoms (VAS 8/10; WOMAC 55; mMRC grade 2), with stable vital parameters. Serum calcium was 6.7 mg/dL. Bone mineral status assessed by quantitative ultrasound (QUS) of the right tibia showed a T-score of -2.6, suggestive of reduced bone density; however, QUS is a screening tool and not definitive for diagnosing osteoporosis.

Renal, liver, and thyroid function tests were within normal limits (Table 1), helping to exclude common secondary causes of bone loss. However, a comprehensive metabolic bone evaluation, including serum phosphate, magnesium, parathyroid hormone (PTH), alkaline phosphatase, and corrected calcium, was not performed due to financial constraints. This may restrict the definitive differentiation between osteoporosis and other metabolic bone disorders, such as osteomalacia.

**Table 1:** Changes in bone mineral density and laboratory parameters during treatment and follow-up ALT, alanine aminotransferase; AST, aspartate aminotransferase; BMD, bone mineral density; BQI, bone quality index; BSL, blood sugar level; ESR, erythrocyte sedimentation rate; KFT, kidney function test; LFT, liver function test; QUS, quantitative ultrasound; SGOT, serum glutamic-oxaloacetic transaminase; SGPT, serum glutamic-pyruvic transaminase; T3, triiodothyronine; T4, thyroxine; TSH, thyroid-stimulating hormone

Parameter	Before treatment	After treatment	Follow-up (February 16, 2026)	Reference range
T-score (QUS)	February 4, 2025	December 1, 2025	-	-
	-2.6 (right tibia)	+0.59 (right tibia), +0.70 (left tibia)	-	> -1 (normal), -1 to -2.5 (osteopenia), ≤ -2.5 (osteoporosis)
BQI	-	117	-	-
Serum calcium (mg/dL)	February 4, 2025	November 26, 2025	10.1	8.5-10.5
	6.7	9.9	-	-
25(OH) Vitamin D (ng/mL)	-	-	17.3	20-50
Blood investigations	April 24, 2025	December 23, 2025	-	-
Hemoglobin (g/dL)	11.9	12.1	-	12-15
Total leukocyte count (/mm³)	4800	5000	-	4000-11,000
Platelet count (lakhs/mm³)	3.37	2.91	-	1.5-4.5
ESR (mm/hr)	44	25	-	<20
KFT	April 24, 2025	December 23, 2025	-	-
Blood urea nitrogen (mg/dL)	26.5	21.3	-	15-40
Serum creatinine (mg/dL)	1.03	0.96	-	0.6-1.2
Uric acid (mg/dL)	4.6	4.3	-	3.5-7.2
Lipid profile	April 24, 2025	December 23, 2025	-	-
Total cholesterol (mg/dL)	234	221	-	<200
Triglycerides (mg/dL)	93.1	78.5	-	<150
BSL	April 24, 2025	December 23, 2025	-	-
Fasting blood sugar (mg/dL)	95	90	-	70-100
Postprandial blood sugar (mg/dL)	108	101	-	<140
LFT	April 24, 2025	December 23, 2025	-	-
Total bilirubin (mg/dL)	0.39	0.37	-	0.3--1.2
Direct bilirubin (mg/dL)	0.13	0.07	-	0.0-0.3
SGOT (AST) (U/L)	34.2	28.6	-	5-40
SGPT (ALT) (U/L)	15.1	18.8	-	5-40
Thyroid profile	-	February 4, 2026	-	-
T3 (ng/dL)	-	112.5	-	60-200
T4 (µg/dL)	-	9.66	-	4.5-12
TSH (µU/mL)	-	2.63	-	0.35-5.50 (18-55 years)

The presence of hypocalcemia and vitamin D deficiency increases the probability of an underlying metabolic bone disorder such as osteomalacia or mixed pathology. At the same time, the low bone density values, along with the history of abdominal hysterectomy and the absence of major secondary risk factors, suggest a possible coexisting osteoporotic component. Therefore, in this case, the diagnosis was considered a metabolic bone disorder with probable osteoporotic involvement, correlating with Asthi-Kshaya (bone tissue depletion) in Ayurveda (Table 1).

Timeline of events

**Figure 3:**
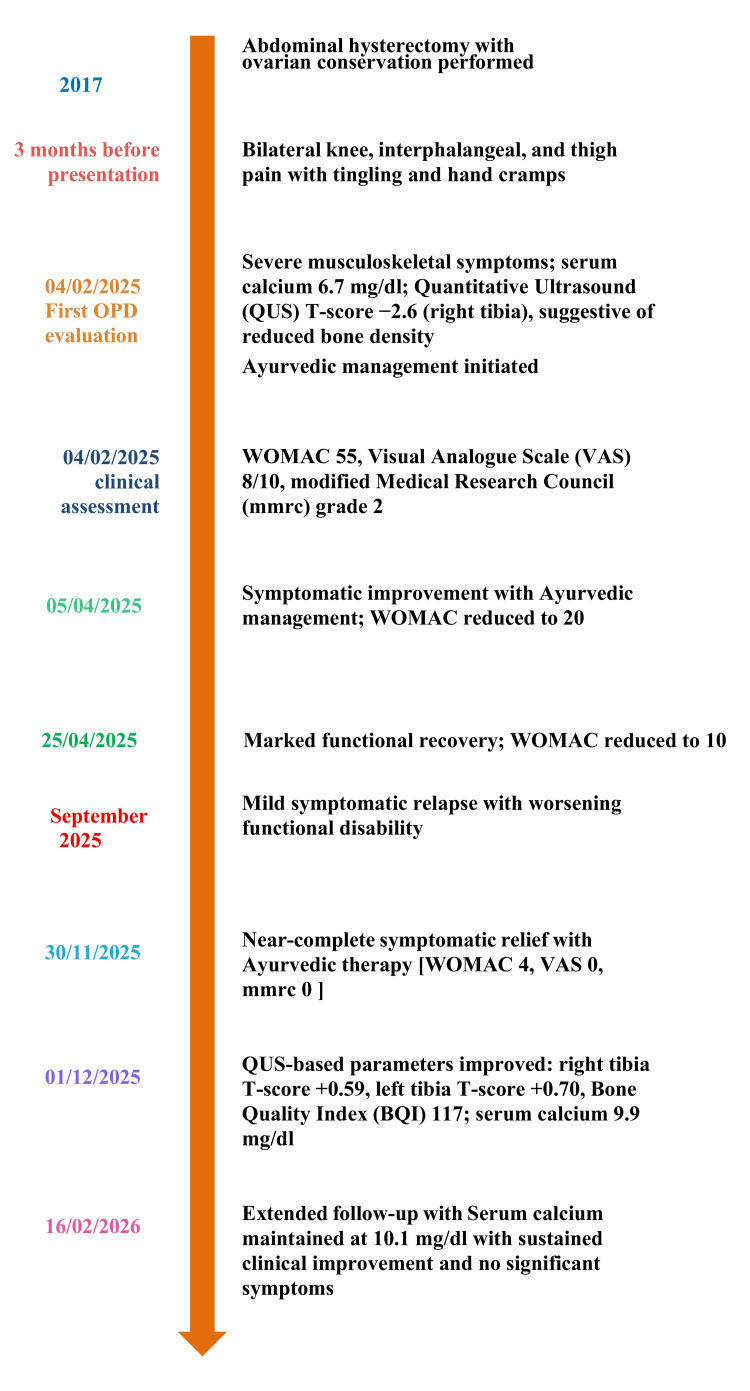
Timeline of events The figure illustrates the chronological sequence of symptom onset, baseline evaluation, integrative Ayurvedic management, follow-up assessments, temporary relapse, and subsequent clinical, biochemical, and QUS-based changes observed during treatment and extended follow-up. mMRC, modified Medical Research Council; QUS, quantitative ultrasound; VAS, Visual Analogue Scale; WOMAC, Western Ontario and McMaster Universities Osteoarthritis Index

The timeline of events is shown in Figure 3.

Therapeutic interventions

The therapeutic interventions are shown in Table 2.

**Table 2:** Therapeutic interventions

Phase	Treatment period	Intervention	Composition/details	Dose per administration	Frequency and timing	Route	Preparation method
Phase 1	February 4, 2025 to April 25, 2025	Combination oral formulation	Ashwagandha churna 1.5 g + Shigrubeej churna 1.5 g + Babul churna 1.5 g + Guduchi satwa 250 mg + Prawal bhasma 250 mg	5 g	Twice daily (BD); morning on an empty stomach and evening before meals	Oral	Ashwagandha churna, Shigrubeej churna, Babul churna, and Guduchi satwa powder preparation method (Churna Kalpana) in Sharangdhara Samhita [8]. Prawal bhasma procured from Dhotpapeshwar Pharmacy, Lic. No.: AYU-150, Batch: P230100018
Phase 2	April 10, 2025 to April 25, 2025	Anuvasan Basti	Guduchyadi taila + (60 mL) Panchtikta ghrita (30 mL) mixed with rock salt	90 mL	Total 12 Basti in Karma Basti manner	Rectal (given after a meal with Basti Yantra)	Guduchyadi taila procured from SNA Oushadhsala Private Limited, Lic. No.: 13/25D/76, Batch: TO680; Panchtikta ghrita procured from Nagarjun Pharmaceuticals, Lic. No.: GA/331, Batch: 398
	April 10, 2025 to April 25, 2025	Mustadi Yapan Basti	Saindhava salt 10 g, honey 100 g, Guduchyadi oil 80 mL, Panchtikta ghrita 80 mL, kalka dravyas (paste) 30 g, milk processed with Mustadi Yapan Basti kwatha (decoction) 300 mL, Majja (goat femur bone marrow) 120 mL	720 mL	Total 6 Basti in Karma Basti manner	Rectal (given before a meal with Basti Yantra)	Basti preparation method according to Charaka Samhita Siddhisthana [9]
Phase 3	September 2025 to November 30, 2025	Combination oral formulation (repeat course)	Ashwagandha churna 1.5 g + Shigrubeej churna 1.5 g + Babul churna 1.5 g + Guduchi satwa 250 mg + Prawal bhasma 250 mg	5 g	Twice daily (BD); morning on an empty stomach and evening before meals	Oral	Ashwagandha churna, Shigrubeej churna, Babul churna, and Guduchi satwa powder preparation method (Churna Kalpana) in Sharangdhara Samhita [8]

**Figure 4:**
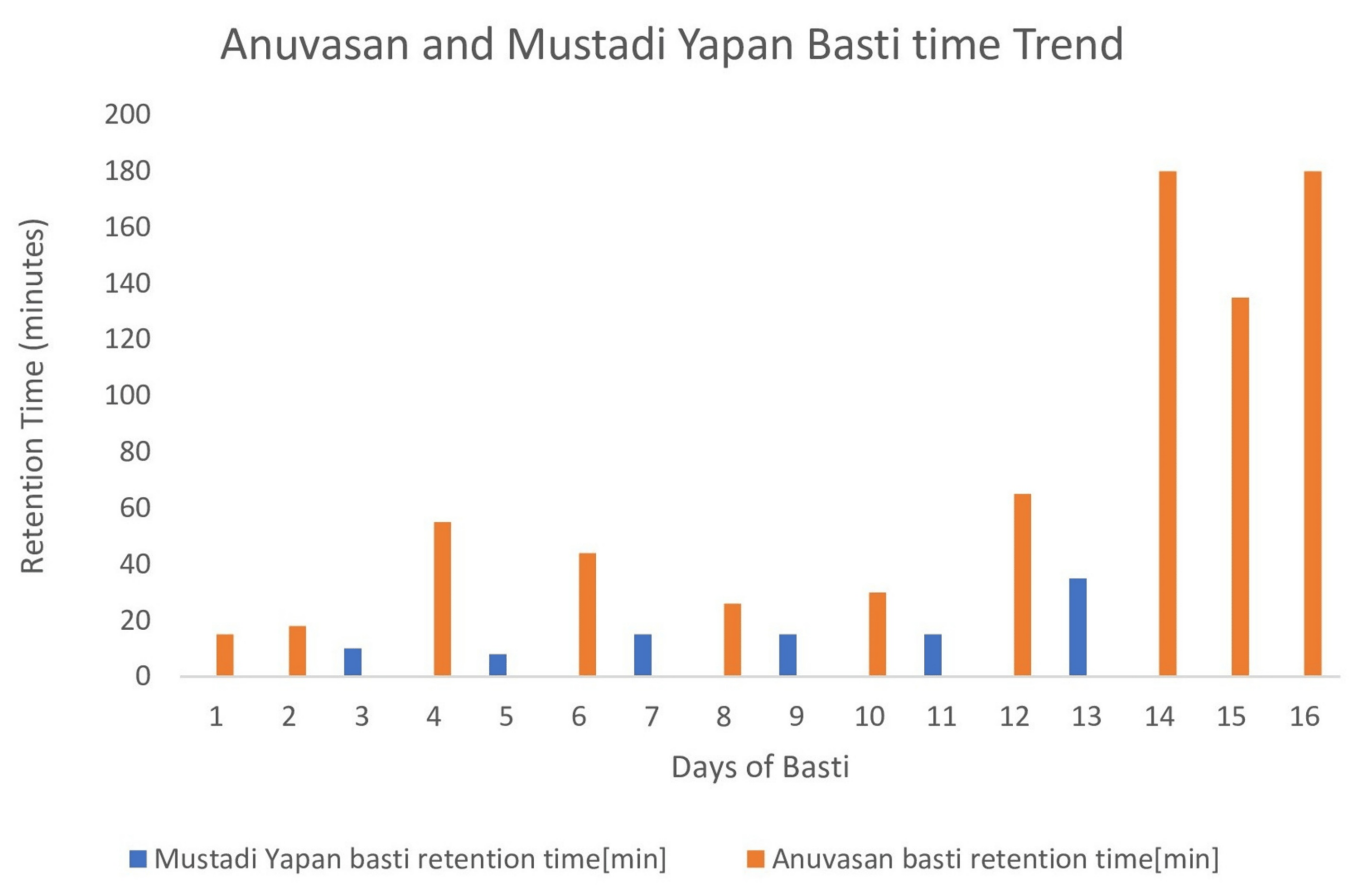
Retention time trend during Anuvasan Basti and Mustadi Yapan Basti administration The figure illustrates day-wise variation in retention time observed during administration of Anuvasan Basti and Mustadi Yapan Basti throughout the treatment period. A progressive increase in retention duration was observed over consecutive treatment sessions, which may reflect improved colonic tolerance, absorptive adaptation, and possible modulation of gut-associated physiological processes linked to mineral balance and bone metabolism.

The retention time of Basti increased over consecutive days. This increase may indicate better colonic absorptive efficiency and mucosal immune regulation. These changes could support modulation of the gut-bone axis linked to bone turnover and mineral balance (Figure 4).

Follow-up and outcomes

The patient was followed through scheduled clinical visits with serial assessment of symptoms, functional status, and laboratory parameters. Standardized outcome measures (VAS and WOMAC scores) showed progressive improvement during the treatment period. A brief interruption in therapy was associated with mild symptom recurrence, which resolved upon resuming treatment. At final follow-up, the patient demonstrated sustained clinical and functional improvement without new adverse events (Figure 1, Figure 2, Table 1).

At extended follow-up (February 16, 2026), conducted after cessation of therapy and without continuation of medication beyond November 30, 2025, blood investigations remained within normal limits, with serum calcium maintained at 10.1 mg/dL, indicating sustained biochemical improvement but not directly reflecting improvement in bone mineral density. QUS-based assessment showed improvement in T-score from -2.6 at baseline to +0.59 at follow-up (right tibia) (Table 1).

## Discussion

The present case of post-hysterectomy osteoporosis highlights the complex interplay of hormonal decline, bone loss, and systemic symptoms. Conventional pharmacotherapy, albeit effective in reducing fracture risk, has limitations, including variable adherence, gastrointestinal and rare skeletal adverse events, and long-term safety concerns. Ayurvedic interventions are increasingly being explored for their potential bone-protective effects via modulation of osteoblastic and osteoclastic activity, anti-inflammatory actions, antioxidant effects, and impacts on bone remodeling pathways. The integrative protocol in this case combined nutritive and lipid-based rectal therapy. It also used multicomponent mineral supplements of botanical origin.

Mustadi Yapan Basti used in this case included milk and bone marrow processed with Tikta rasa (bitter taste) and Madhura rasa (sweet taste) herbs, which are relevant to skeletal metabolism [9]. Rectal and enteric lipid-based delivery systems may enhance systemic bioavailability of lipophilic phytoconstituents and could influence gut-associated immune and metabolic processes. These effects are discussed within the conceptual framework of the gut-bone axis, although direct evidence in this context remains limited. This framework describes the relationships among intestinal absorption efficiency, mucosal immune activity, microbiome metabolites, and inflammatory tone, which contribute directly to skeletal remodeling and mineral homeostasis [10,11].

Dalhana describes a functional relationship between Purishadhara kala (the colonic functional layer) and Asthidhara kala (the bone-supportive layer) [12]. This conceptual linkage has been discussed in light of emerging evidence suggesting potential interactions among colonic mucosal health, immune signaling, and bone metabolism. Some studies indicate that intestinal immune signaling may influence bone turnover, calcium utilization, and osteo-immunologic balance, thereby providing a possible biological link between enteric processes and skeletal outcomes; however, these associations remain exploratory and were not directly evaluated in the present case [13,14].

Anuvasana Basti was administered using Panchtikta ghrita (a ghee-based bitter polyherbal formulation) and Guduchyadi Taila (medicated oil) [15,16]. Panchtikta ghrita and Guduchyadi Taila contain bitter-dominant herbs and have been reported to exhibit anti-inflammatory, antioxidant, and bone-protective activities. A notable feature of the regimen is the predominance of bitter herbs. Emerging evidence suggests that bitter taste receptors (type 2 taste receptors, T2Rs) are expressed in extra-oral tissues, including mesenchymal and osteoblast-lineage cells, where they may participate in intracellular calcium signaling, mitogen-activated protein kinase pathway activity, and inflammatory modulation. Experimental findings indicate that T2R activation could influence osteoblast differentiation and cellular stress responses, suggesting a possible receptor-level relevance of bitter-dominant phytotherapeutic strategies in bone metabolism [17,18].

The polyherbomineral supplementation is mechanistically supported by evidence that *Moringa oleifera*, *Withania somnifera*, *Tinospora cordifolia*, and *Acacia arabica *promote osteogenesis and reduce bone resorption [19-22]. At the same time, coral-derived calcium (Prawal bhasma) improves calcium balance and bone microarchitecture in experimental models [23].

This case report shows concurrent clinical and biochemical improvement, with changes in QUS-based parameters, following a structured integrative therapeutic protocol; however, these findings are observational and should be interpreted cautiously given the single-case design and the use of a screening modality. The observed clinical and functional improvements may be associated with the proposed gut-bone-immune and receptor-related mechanisms, but these remain hypothesis-generating and require validation through well-designed controlled studies to establish safety, reproducibility, and clinical utility in osteoporosis management.

VAS, WOMAC, and mMRC scores were used to assess symptomatic and functional changes; however, these measures are not specific to osteoporosis and should be interpreted as supportive indicators of clinical improvement rather than direct measures of bone health.

No adverse events were observed during the treatment period. Bone mineral status was assessed using QUS, a screening modality not equivalent to dual-energy X-ray absorptiometry (DXA), which could not be performed due to financial constraints. A comprehensive metabolic bone evaluation, including albumin-corrected or ionized calcium, serum phosphate, magnesium, PTH, and alkaline phosphatase, was not performed due to financial constraints, limiting definitive etiological classification and differentiation from conditions such as osteomalacia. Baseline vitamin D deficiency was documented clinically but could not be fully reported due to the unavailability of the initial laboratory record; only follow-up vitamin D levels were available. Therefore, the findings are observational and hypothesis-generating, requiring validation through well-designed controlled studies.

## Conclusions

This case describes symptomatic and biochemical improvement in a patient receiving an Ayurvedic intervention, with concurrent changes in QUS-based parameters. However, these findings are derived from a single case and a screening modality; hence, despite cautious interpretation, this treatment generates a research question regarding the evaluation of the Ayurveda-based approach for improvement in bone mineral density or underlying mechanisms. The observations are hypothesis-generating and warrant further investigation using standardized interventions, DXA-based assessment, comprehensive metabolic evaluation, and controlled study designs to establish clinical efficacy, safety, and biological relevance.
